# Performance of the Osteoporosis Self-Assessment Tool for Asians (OSTA) Index for Osteoporosis Screening in Thai Men: A Cross-Sectional Study

**DOI:** 10.7759/cureus.79789

**Published:** 2025-02-27

**Authors:** Jirapong Leeyaphan, Karn Rojjananukulpong, Piyapong Intarasompun, Yuthasak Peerakul

**Affiliations:** 1 Department of Disease Control, Bamrasnaradura Infectious Diseases Institute, Nonthaburi, THA

**Keywords:** bone mineral density, men, osteoporosis, osteoporosis self-assessment tool for asians (osta), sensitivity, specificity, thailand

## Abstract

Background

Addressing the under-recognition of osteoporosis in men necessitates the use of suitable screening tools tailored to the male population. The Osteoporosis Self-Assessment Tool for Asians (OSTA) index is a reliable tool for evaluating the risk of osteoporosis. This cross-sectional study aims to assess the predictive performance of the OSTA index in Thai men. Additionally, the study compares the performance of the OSTA index with the Khon Kaen Osteoporosis Study (KKOS) score and an age cutoff value of ≥70 years.

Methods

Between November 2017 and October 2024, men aged 50 years or older who underwent dual-energy X-ray absorptiometry and did not receive any osteoporosis treatment were included. The performance of the individual OSTA index cutoff values in predicting osteoporosis was assessed.

Results

A total of 427 men were included in this study and classified into normal bone mineral density (BMD) (175 men, 41%), osteopenia (210 men, 49.2%), and osteoporosis (42 men, 9.8%). The mean age, weight, and body mass index were 76.4 years, 65.9 kg, and 24.1 kg/m², respectively. The OSTA index cutoff of −1 at any BMD site produced a Youden index of 0.187, with a sensitivity of 85.7% and a specificity of 33%. Meanwhile, the OSTA index cutoff of −4 at any BMD site resulted in a Youden index of 0.395, with a sensitivity of 59.5% and a specificity of 80%. The area under the receiver operating characteristic curve (AUC) of the KKOS score ≤−1 was 0.636. The AUC of the OSTA index ≤−1 was 0.594, which was significantly lower than the AUC of the KKOS score ≤−1 (P = 0.003). The AUC of age ≥70 years was 0.494, which was significantly lower than the AUC of the OSTA index ≤−1 (P < 0.001).

Conclusion

For osteoporosis screening in Thai men, the OSTA index cutoff value of −1 is more appropriate than a cutoff value of −4. However, the KKOS score cutoff value of ≤−1 serves as the most effective predictor for osteoporosis screening.

## Introduction

Osteoporosis is a silent systemic skeletal disease that affects women more often than men [1]. The literature reported that the prevalence of osteoporosis in men within Asian-Pacific countries varies significantly, ranging from 5% to over 40%, depending on the age range or specific age groups considered in different countries [2]. The prevalence of osteoporosis in men was observed to be lower compared to that in women. While the overall prevalence of fragility fractures is higher in women than in men, men experience a greater reduction in life expectancy, particularly following hip fractures. For every six women and every three men who experienced a hip fracture, there was one additional death compared to the expected rate in the general population [3]. Moreover, male hip fracture patients have a higher mortality rate than female patients, even though men are, on average, four years younger at the time of fracture [4]. The under-recognition of osteoporosis in men could be addressed using appropriate screening tools tailored to the male population.

Risk assessment tools have recently been reviewed for their use in Asian-Pacific countries. Risk assessment tools were categorized into fracture risk and osteoporosis risk tools. For example, the Fracture Risk Assessment Tool (FRAX®) was the most commonly recommended tool in fracture risk assessment, and the Male Osteoporosis Risk Estimation Score (MORES) is a clinical tool used to assess a man's risk of developing osteoporosis by considering factors such as age, weight, and a history of chronic obstructive pulmonary disease [5]. The Osteoporosis Self-Assessment Tool for Asians (OSTA) index is a reliable tool for assessing the risk of osteoporosis. It was developed using data from women in selected Asian countries [6]. Despite the OSTA index utilizing only body weight and age as potential predictors for osteoporosis in women, it was widely utilized as a screening tool for osteoporosis prior to confirming the diagnosis with dual-energy X-ray absorptiometry (DXA) in the osteoporosis clinical practice guidelines (CPG) [5], while its performance was still validated in men across various countries globally, not just to Asian countries [7-12]. As a result, research has shown that the OSTA index exhibits varying levels of sensitivity and specificity across different male or female populations.

Nowadays, there has been an increase in the number of DXA machines in many countries [13]. The osteoporosis CPGs still recommend that individuals with specific clinical risk factors for osteoporosis or fragility fractures be considered for evaluation before a confirmed diagnosis of DXA [5]. In Thailand, the Khon Kaen Osteoporosis Study (KKOS) score has been developed for Thai postmenopausal women and used for identifying osteoporosis risk. This tool was more sensitive and specific than the OSTA index when it was validated in Thai women [14]. The KKOS score was also validated in Thai men. The KKOS score, with a cutoff point of ≤−1, demonstrated an overall sensitivity of 72.5% and specificity of 73.2% [15]. However, the utility of the KKOS score was not directly comparable to the OSTA index performance in predicting low bone mineral density (BMD) in Thai men.

According to the osteoporosis CPG of Thailand, it is recommended that all men aged 70 years or older should be diagnosed using DXA. This recommendation aligns with the osteoporosis CPGs or recommendations from other Asian-Pacific countries [16-18]. However, there was a study mentioning increased cost and lack of evidence supporting the performance of solely age ≥70 years as the predictor for osteoporosis [19]. Therefore, the performance of age ≥70 years in predicting osteoporosis could be also evaluated.

The present study aimed to evaluate the prediction performance for osteoporosis of the OSTA index in Thai men. In addition, the performance of the OSTA index was also compared with the KKOS score and the age ≥70-year cutoff value.

## Materials and methods

Data collection and study population

Demographic and clinical data, including age, weight, height, and underlying diseases, were obtained and analyzed from electronic medical records and the DXA machine database of men aged 50 and older who underwent DXA examinations between November 2017 and October 2024 in Bamrasnaradura Infectious Diseases Institute, Nonthaburi, Thailand.. Participants who had received prior osteoporosis treatment before BMD measurement or had a history of secondary osteoporosis-related conditions (such as chronic kidney disease, end-stage renal disease, malignancy, hyperthyroidism, or rheumatoid arthritis) were excluded from the study. Additionally, participants with a history of lumbar spine or hip surgery were also excluded. The study protocol received approval from the Institutional Review Board of the Bamrasnaradura Infectious Diseases Institute (approval number: S042h/67_ExPD).

BMD measurement

The participants' BMD was measured using the same DXA machine (Discovery W; Hologic, Inc., Bedford, MA, USA) at the said hospital throughout the data collection period. For individuals with multiple densitometry records, only the first measurement for each person was included. A professionally trained technician regularly assessed the DXA machine using the spine phantom to ensure system stability and optimal performance. The interpretation of T-scores obtained from DXA scans at two anatomical sites, the lumbar spine (L1-4) and the hip (femoral neck and total hip), adheres to the criteria established by the World Health Organization. Osteoporosis is defined by T-scores of ≤−2.5 standard deviation (SD), and osteopenia is characterized by T-scores ranging from −1.0 to −2.5 SD, while normal bone density is indicated by T-scores of ≥−1.0 SD.

Weight and height measurement

Each participant's body weight and height were recorded on the day of the BMD assessment. Participants wore lightweight clothing and removed their shoes for weight and height measurements. In this study, a weighing scale with a precision of 0.1 kg and a height scale with an accuracy of 1 cm were utilized. Height and weight measurements were obtained using an electronic stadiometer (NAGATA BW-1116MH; Nagata Scale Co., Ltd., Tainan, Taiwan).

The OSTA index and the KKOS score calculation

The OSTA index was calculated using the formula 0.2 × (body weight − age), with the decimals truncated to an integer. The KKOS score was calculated for each man by using the summation of age and weight score from the KKOS score table [14].

Statistical analysis

The participants were stratified into three distinct groups according to their highest baseline BMD T-score across the three evaluated BMD sites: normal, osteopenia, and osteoporosis. Participants were also categorized into two groups: one group with participants having a T-score of ≤−2.5 (osteoporosis group) and another group with participants having a T-score of >−2.5 (non-osteoporosis group). Descriptive statistics, such as means with SDs or medians with interquartile ranges, were presented based on data distribution. Body mass index (BMI) is calculated by dividing weight in kilograms by square height in meters.

The relationship between the OSTA index and T-score was assessed through the Pearson correlation analysis. The discriminatory capabilities of the OSTA index, the KKOS score, and the age ≥70-year cutoff value in identifying osteoporosis were assessed by calculating the area under the receiver operating characteristic curve (AUC) with a 95% confidence interval (CI). The optimal discriminative performance of the OSTA index cutoff values was determined using the maximum value of the Youden index (sensitivity + specificity − 1) [20]. Sensitivity, specificity, positive predictive values, and negative predictive values were analyzed and reported. The AUC of each screening tool was compared for comprehensive evaluation.

To achieve the desired sensitivity or specificity of the OSTA index, an osteoporosis prevalence of 12.6% [21] and a 10% margin of error, an 84% sensitivity, and a 48.6% specificity [22] were targeted based on 95% CI. As a result, the sample sizes needed to reach the sensitivity and specificity targets were calculated to be 410 and 110 participants, respectively [23]. Hence, the study incorporated at least a sample size of 410 participants to ensure the attainment of research objectives. The statistical analyses were performed using STATA version 15 (StataCorp LLC, College Station, TX, USA), and a P-value less than 0.05 was regarded as statistically significant.

## Results

**Figure 1:**
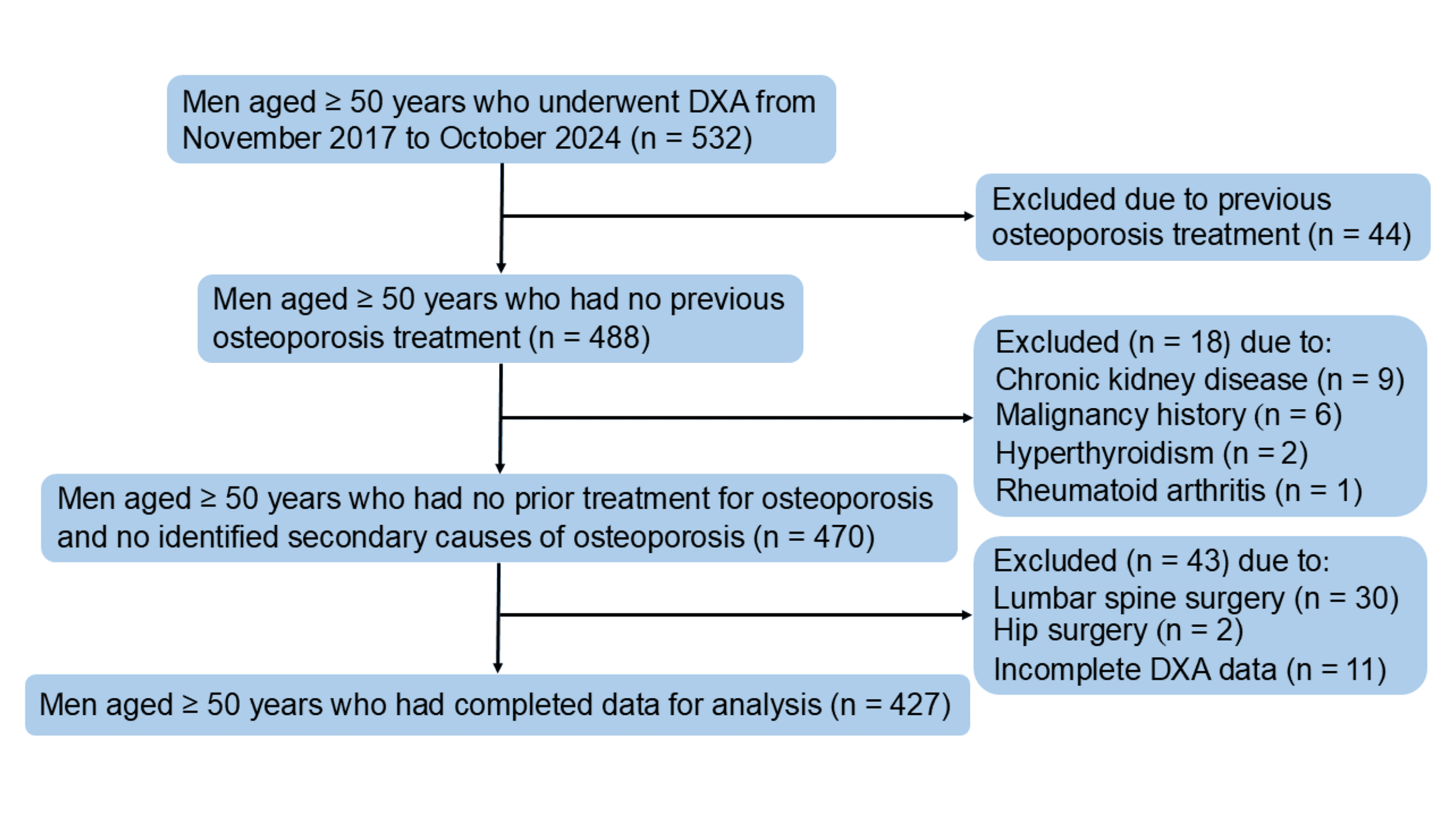
Participants' enrollment flowchart DXA: dual-energy X-ray absorptiometry

This study included 532 men aged 50 years and older. Of these, exclusions were made for the following reasons: 44 participants due to prior osteoporosis treatment, nine due to chronic kidney disease, six due to a history of malignancy, two due to hyperthyroidism, one due to rheumatoid arthritis, 32 due to previous spinal or hip surgery, and 11 due to incomplete DXA data. As a result, the final analysis comprised 427 men (Figure 1).

Prevalence of osteoporosis

The prevalence of osteoporosis, osteopenia, and normal BMD at any site were 42 (9.8%), 210 (49.2%), and 175 (41%), respectively. The mean age, weight, and BMI were 76.4 years, 65.9 kg, and 24.1 kg/m², respectively. The OSTA index and KKOS score mean were −1.85 and −1.34. The clinical characteristics of the participants are summarized in Table 1.

**Table 1:** Demographic and clinical characteristics of the study participants Values are expressed as mean ± standard deviation or number (percentage) BMI: body mass index; OSTA: Osteoporosis Self-Assessment Tool for Asians; KKOS: Khon Kaen Osteoporosis Study; BMD: bone mineral density

Variables	Value	Range
Age (years)	76.4 ± 6.7	53 to 95
Age group (years)		
50-69	46 (10.8)	
70-89	371 (86.9)	
≥90	10 (2.3)	
Height (cm)	165.3 ± 6.2	147 to 186
Weight (kg)	65.9 ± 10.6	37 to 105
BMI (kg/m^2^)	24.1 ± 3.4	15.1 to 35.7
BMI group (kg/m^2^)		
<18.5	20 (4.7)	
18.5-22.9	139 (32.6)	
23-24.9	102 (23.9)	
≥25	166 (38.9)	
OSTA index	−1.85 ± 2.44	−11 to 6
KKOS score	−1.34 ± 4.91	−17.5 to 10.5
BMD (g/cm^2^)		
Lumbar spine	1.05 ± 0.20	0.61 to 1.77
Femoral neck	0.71 ± 0.13	0.34 to 1.18
Total hip	0.88 ± 0.14	0.46 to 1.43
BMD (T-score)		
Lumbar spine	0.18 ± 1.69	−3.5 to 6.2
Femoral neck	−1.10 ± 1.04	−4.1 to 2.7
Total hip	−0.45 ± 1.01	−3.5 to 3.6
Diagnosis		
Normal	175 (41.0)	
Osteopenia	210 (49.2)	
Osteoporosis	42 (9.8)	

Performance of the OSTA index cutoff values

**Figure 2:**
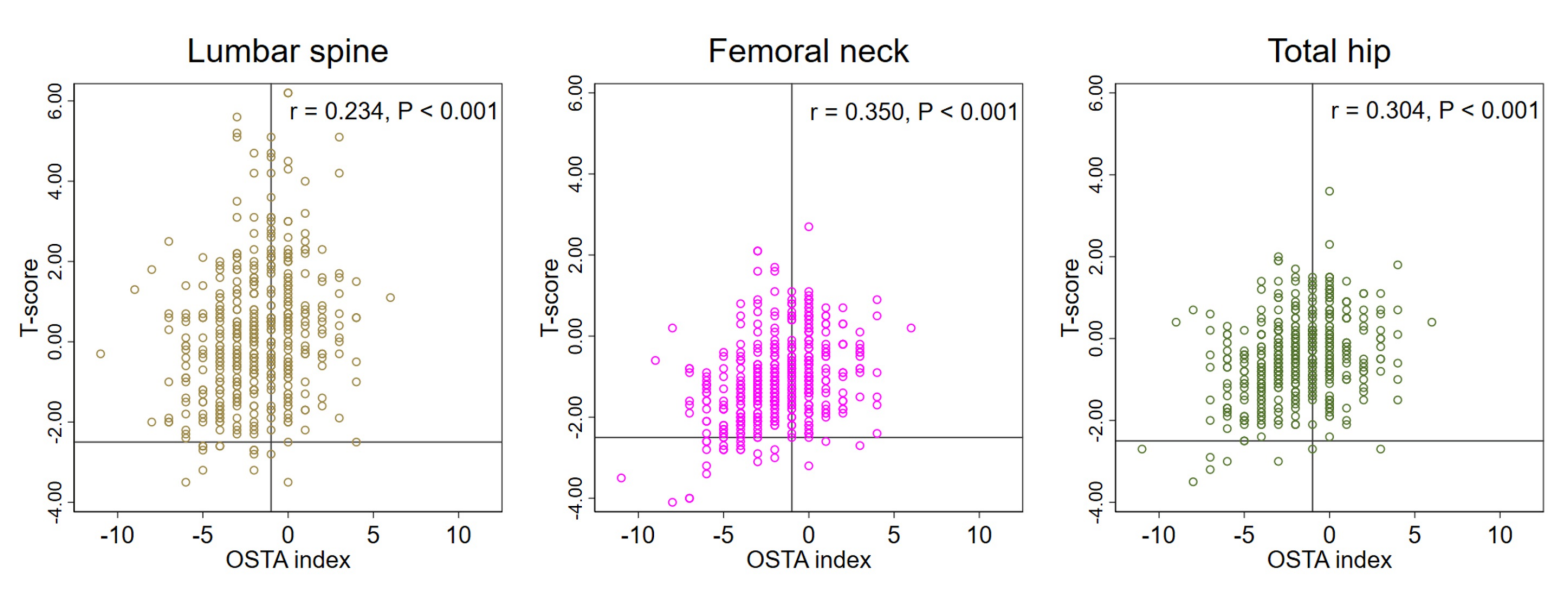
T-score distribution by OSTA index OSTA: Osteoporosis Self-Assessment Tool for Asians; r: Pearson correlation coefficient

The correlation coefficient between the OSTA index and T-score of the lumbar spine, femoral neck, and total hip DXA was 0.234, 0.350, and 0.304, respectively (all P < 0.001) (Figure 2).

The OSTA index cutoff values for osteoporosis prediction at all BMD sites were suggested as the OSTA index cutoff −4 was an optimal OSTA index cutoff value according to the maximum Youden index. The OSTA index cutoff −4 at total hip BMD yielded a Youden index of 0.472, with a sensitivity of 70%, specificity of 77.2%, positive predictive value of 6.9%, and negative predictive value of 99.1%. The OSTA index cutoff −4 at any BMD site provided a Youden index of 0.395, with a sensitivity of 59.5%, specificity of 80%, and positive predictive value of 24.5% (Table 2).

**Table 2:** The OSTA index cutoff value performance in predicting osteoporosis at various BMD sites OSTA: Osteoporosis Self-Assessment Tool for Asians; BMD: bone mineral density; AUC: area under the receiver operating characteristic curve; CI: confidence interval; PPV: positive predictive value; NPV: negative predictive value; LR+: positive likelihood ratio

Site of BMD	OSTA index cutoff	AUC (95% CI)	Sensitivity (%)	Specificity (%)	PPV (%)	NPV (%)	LR+	Youden index
Femoral neck	−4	0.719 (0.631-0.807)	64.5	79.3	19.6	96.6	3.12	0.438
	−3	0.708 (0.629-0.786)	77.4	64.1	14.5	97.3	2.16	0.415
	−2	0.654 (0.584-0.724)	83.9	47.0	11.0	97.4	1.58	0.309
	−1	0.598 (0.534-0.663)	87.1	32.6	9.2	97.0	1.11	0.197
	0	0.537 (0.490-0.584)	93.5	13.9	7.8	96.5	1.09	0.074
	1	0.524 (0.490-0.559)	96.8	8.1	7.6	97.0	1.05	0.049
	2	0.505 (0.472-0.538)	96.8	4.3	7.3	94.4	1.01	0.011
	3	0.509 (0.502-0.515)	100.0	1.8	7.4	100.0	1.02	0.018
	4	0.501 (0.499-0.504)	100.0	0.3	7.3	100.0	1.00	0.003
Total hip	−4	0.736 (0.585-0.887)	70.0	77.2	6.9	99.1	3.07	0.472
	−3	0.711 (0.578-0.843)	80.0	62.1	4.8	99.2	2.11	0.421
	−2	0.627 (0.494-0.759)	80.0	45.3	3.4	99.0	1.46	0.253
	−1	0.608 (0.508-0.709)	90.0	31.7	3.1	99.2	1.32	0.217
	0	0.517 (0.418-0.617)	90.0	13.4	2.4	98.2	1.04	0.034
	1	0.488 (0.390-0.587)	90.0	7.7	2.3	97.0	0.97	−0.023
	2	0.470 (0.372-0.569)	90.0	4.1	2.2	94.4	0.94	−0.059
	3	0.508 (0.502-0.515)	100.0	1.7	2.4	100.0	1.02	0.017
	4	0.501 (0.499-0.504)	100.0	0.2	2.3	100.0	1.00	0.002
Lumbar spine	−4	0.636 (0.508-0.764)	50.0	77.1	7.8	97.5	2.19	0.271
	−3	0.558 (0.429-0.686)	50.0	61.6	4.8	96.9	1.30	0.116
	−2	0.603 (0.490-0.715)	75.0	45.5	5.1	97.9	1.38	0.205
	−1	0.564 (0.463-0.666)	81.3	31.6	4.4	97.7	1.19	0.129
	0	0.537 (0.473-0.600)	93.8	13.6	4.1	98.2	1.09	0.074
	1	0.508 (0.445-0.570)	93.8	7.8	3.8	97.0	1.02	0.016
	2	0.489 (0.427-0.551)	93.8	4.1	3.7	94.4	0.98	−0.021
	3	0.476 (0.415-0.538)	93.8	1.5	3.6	85.7	0.95	−0.047
	4	0.501 (0.499-0.504)	100.0	0.2	3.8	100.0	1.00	0.002
Any site	−4	0.698 (0.620-0.775)	59.5	80.0	24.5	94.8	2.98	0.395
	−3	0.667 (0.593-0.742)	69.0	64.4	17.5	95.0	1.94	0.334
	−2	0.642 (0.577-0.708)	81.0	47.5	14.4	95.8	1.54	0.285
	−1	0.594 (0.535-0.652)	85.7	33.0	12.2	95.5	1.28	0.187
	0	0.534 (0.491-0.577)	92.9	14.0	10.5	94.7	1.08	0.069
	1	0.517 (0.481-0.552)	95.2	8.1	10.2	93.9	1.04	0.033
	2	0.497 (0.463-0.531)	95.2	4.2	9.8	88.9	0.99	−0.006
	3	0.496 (0.472-0.520)	97.6	1.6	9.8	85.7	0.99	−0.008
	4	0.501 (0.499-0.504)	100.0	0.3	9.9	100.0	1.00	0.003

Additionally, age was divided into two groups: in the ≥70-year age group, the OSTA index cutoff −1 at any BMD site yielded a sensitivity of 94.6% and a specificity of 28.2%, while in the 50-69-year age group, the OSTA index cutoff −1 at any BMD site yielded a sensitivity of 80% and a specificity of 26.8% (Table 3).

**Table 3:** Performance of the selected OSTA index at any BMD site according to age group OSTA: Osteoporosis Self-Assessment Tool for Asians; BMD: bone mineral density; CI: confidence interval

Age group (years)	OSTA index	Sensitivity (%)	95% CI	Specificity (%)	95% CI
50-69	≤−1	80.0	28.4-99.5	26.8	14.2-42.9
	≤−4	20.0	0.5-71.6	100.0	91.4-100.0
≥70	≤−1	94.6	81.8-99.3	28.2	23.5-33.3
	≤−4	64.9	47.5-79.8	77.6	72.8-81.9

**Figure 3:**
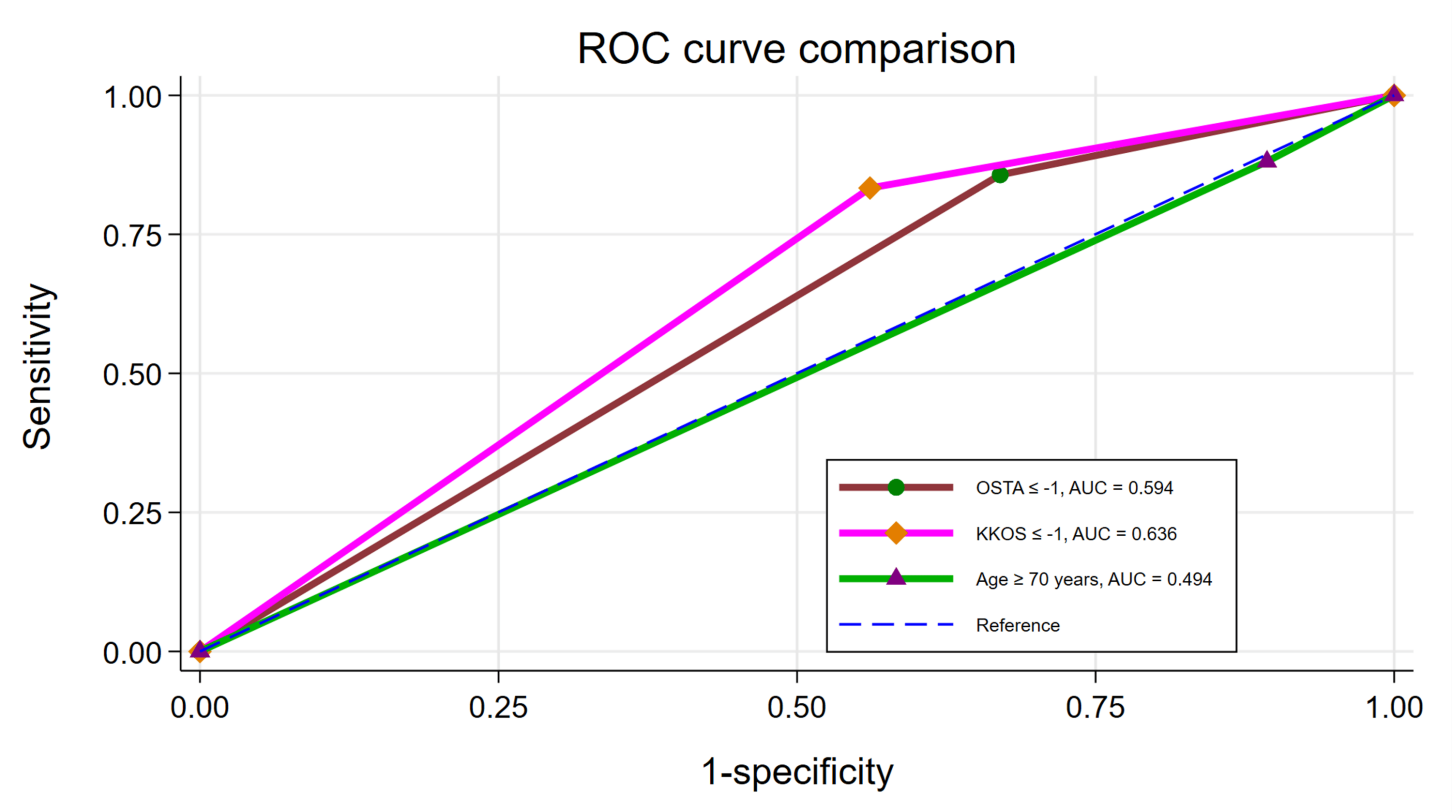
ROC curve comparison ROC: receiver operating characteristic; AUC: area under the receiver operating characteristic curve; OSTA: Osteoporosis Self-Assessment Tool for Asians; KKOS: Khon Kaen Osteoporosis Study

The AUC of the OSTA index ≤−1 was 0.594 which was significantly lower than the AUC of KKOS score ≤−1 (P = 0.003), while the AUC of the OSTA index ≤−1 was significantly higher than the AUC of age ≥70 years (P < 0.001) (Table 4) (Figure 3).

**Table 4:** Comparison of the selected OSTA index with other predictors AUC: area under the receiver operating characteristic curve; CI: confidence interval; OSTA: Osteoporosis Self-Assessment Tool for Asians; KKOS: Khon Kaen Osteoporosis Study

Predictors	AUC (95% CI)	P-value	Sensitivity (%)	95% CI	Specificity (%)	95% CI
OSTA ≤−1 (reference)	0.594 (0.535-0.652)		85.7	71.5-94.6	33.0	28.3-37.9
KKOS ≤−1	0.636 (0.574-0.698)	0.003	83.3	68.6-93.0	43.9	38.9-49.0
Age ≥70 years	0.494 (0.442-0.546)	<0.001	88.1	74.4-96.0	10.6	7.8-14.2
OSTA ≤−4 (reference)	0.698 (0.620-0.775)		59.5	43.3-74.4	80.0	75.7-83.9
KKOS ≤−1	0.636 (0.574-0.698)	0.083	83.3	68.6-93.0	43.9	38.9-49.0
Age ≥70 years	0.494 (0.442-0.546)	<0.001	88.1	74.4-96.0	10.6	7.8-14.2

**Figure 4:**
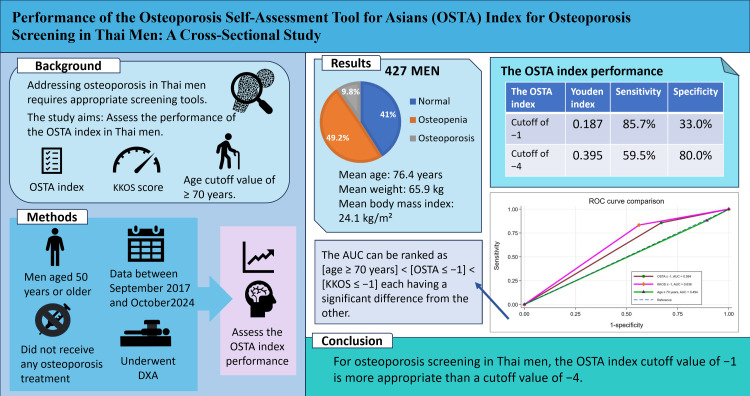
Graphical abstract of the present study OSTA: Osteoporosis Self-Assessment Tool for Asians; KKOS: Khon Kaen Osteoporosis Study; DXA: dual-energy X-ray absorptiometry; ROC: receiver operating characteristic; AUC: area under the receiver operating characteristic curve Image Credits: Naphat Leeyaphan

Figure 4 presents the graphical abstract of the present study.

## Discussion

Figure 4 presents a comprehensive summary of the study's methodology and conclusions. Osteoporosis screening tools have been developed to predict or select people who have osteoporosis risk for appropriate osteoporosis management. Many studies in varying countries tried to develop and validate screening tools for increasing osteoporosis prediction performance. Former osteoporosis screening tools have been developed for postmenopausal women who have a higher osteoporosis prevalence than men. Consequently, there were few studies directly developed for osteoporosis screening tools in men. Moreover, validation studies of postmenopausal women osteoporosis screening tools to evaluate performance in the men group had not been seen frequently. This present study demonstrated the performance of the OSTA index cutoff value when generalized for osteoporosis screening purposes in Thai men. Additionally, the performance of optimal OSTA index cutoff value was compared with the osteoporosis screening tool (the KKOS score) and common primary osteoporosis screening risk for DXA evaluation (age ≥70 years) in the osteoporosis CPG.

Body weight and BMI have been identified as potential factors for osteoporosis [17]. This present study reported a higher mean body weight and BMI compared to the previous study, with values of 65.9 kg versus 62.1 kg and 24.1 kg/m² versus 23.1 kg/m², respectively. As a result, the prevalence of men's osteoporosis in this study is slightly lower than in the previous study (42 (9.8%) versus 52 (12.6%)) [21]. This study evaluated the performance of various OSTA index cutoffs for osteoporosis screening in Thai men. This study demonstrated a value tradeoff between sensitivity and specificity in screening postmenopausal women when using the OSTA index cutoff applied to men. Therefore, a high OSTA index cutoff value demonstrates high sensitivity and low specificity. In contrast, a low OSTA index cutoff value exhibits lower sensitivity compared to a higher cutoff value. Notably, one study indicated that a sensitivity of 90% is appropriate for the OSTA index in the context of osteoporosis screening [24]. The OSTA index cutoff value was the key point of utility selection. Therefore, a sensitivity of 85.7% for the OSTA index −1 cutoff value, as commonly referenced in many kinds of literature [25], may be more suitable compared to a sensitivity of 59.5% for the OSTA index −4 cutoff value when used in screening scenarios. This study demonstrated that for individuals aged 70 years and older, an OSTA index cutoff of −1 at any BMD site resulted in a sensitivity of 94.6%, which is higher than the sensitivity of 85.7% when using the OSTA index −1 cutoff value for the overall age group. Therefore, applying the OSTA index −1 cutoff value within specific age groups may be more advantageous than using the OSTA index −1 cutoff value alone in osteoporosis screening. The impact of age distribution on the performance of osteoporosis screening tools should be elucidated in future studies.

Many studies have attempted to determine the optimal OSTA index cutoff value for identifying individuals at risk of osteoporosis [25]. This study is the first to evaluate the performance of the OSTA index for predicting osteoporosis in Thai men while directly comparing its predictive accuracy with the KKOS score and an age threshold of ≥70 years. This study assessed the performance of various OSTA index cutoff values and selected the most suitable one, comparing it with the KKOS score and the age cutoff value of ≥70 years using the Youden index. The OSTA index with a cutoff value of −1 exhibited a lower AUC compared to the KKOS score with a cutoff value of ≤−1 (0.594 versus 0.636; P = 0.003). Conversely, the OSTA index with a cutoff value of −4 presented a higher AUC in comparison to the KKOS score cutoff value, though this difference was not statistically significant (0.698 versus 0.636; P = 0.083). Additionally, the age cutoff value of ≥70 years demonstrated the lowest AUC when compared to both the OSTA index and the KKOS score cutoff values. Therefore, utilizing the KKOS score cutoff value may be more suitable for osteoporosis screening in Thailand. Future studies are needed to evaluate the performance of the KKOS score and the OSTA index in combination with other risk factors to develop a more predictive model for osteoporosis screening. Additionally, future research should evaluate the cost-effectiveness of osteoporosis screening tools to determine the efficiency of these strategies based on the sex and age demographics of individual countries.

This study has certain limitations. First, it assessed only one population in a tertiary care hospital setting. The results may vary when validated in different populations or settings. Second, this study aims to evaluate the effectiveness of specific tools or predictors in determining the performance of osteoporosis screening. It is important to note that this does not assess fracture risk nor does it provide guidance for treatment decisions based on these clinical factors.

## Conclusions

This study elucidated the performance of the OSTA index in identifying osteoporosis among Thai men. It was found that the OSTA index cutoff value of −1 is more suitable for osteoporosis screening than a cutoff value of −4. However, the KKOS score cutoff value of ≤−1 demonstrated a higher ability to discriminate osteoporosis compared to the OSTA index cutoff value of −1. Additionally, an age cutoff value of ≥70 years showed the lowest discriminative ability for osteoporosis when compared to both the OSTA index cutoff value of −1 and the KKOS score cutoff value of ≤−1.
